# From acute SARS-CoV-2 infection to pulmonary hypertension

**DOI:** 10.3389/fphys.2022.1023758

**Published:** 2022-12-19

**Authors:** Emmanuel Eroume À Egom, Haaris A. Shiwani, Brice Nouthe

**Affiliations:** ^1^Institut du Savoir Montfort (ISM), University of Ottawa, Ottawa, ON, Canada; ^2^ CIEL, Centre d’Innovation et de Commercialisation en Recherche Clinique et Bio-Médicale Immânow’EL, Béatitude/Nkolbisson, Yaoundé, Cameroon; ^3^ Laboratory of Endocrinology and Radioisotopes, Institute of Medical Research and Medicinal Plants Studies (IMPM), Yaoundé, Cameroon; ^4^ Burnley General Hospital, East Lancashire Hospitals NHS Trust, Burnley, United Kingdom; ^5^ Department of Medicine, The University of British Columbia, Vancouver, BC, Canada

**Keywords:** COVID-19, pulmonary arterial hypertension, chronic thromboembolic pulmonary hypertension, SARS-COV-2, vascular disease, SARS-COV-2 infection, Natriuretic Peptide Receptor type C (NPR-C)

## Abstract

As the world progressively recovers from the acute stages of the coronavirus disease 2019 (COVID-19) pandemic, we may be facing new challenges regarding the long-term consequences of COVID-19. Accumulating evidence suggests that pulmonary vascular thickening may be specifically associated with COVID-19, implying a potential tropism of severe acute respiratory syndrome coronavirus 2 (SARS-COV-2) virus for the pulmonary vasculature. Genetic alterations that may influence the severity of COVID-19 are similar to genetic drivers of pulmonary arterial hypertension. The pathobiology of the COVID-19-induced pulmonary vasculopathy shares many features (such as medial hypertrophy and smooth muscle cell proliferation) with that of pulmonary arterial hypertension. In addition, the presence of microthrombi in the lung vessels of individuals with COVID-19 during the acute phase, may predispose these subjects to the development of chronic thromboembolic pulmonary hypertension. These similarities raise the intriguing question of whether pulmonary hypertension (PH) may be a long-term sequela of SARS-COV-2 infection. Accumulating evidence indeed support the notion that SARS-COV-2 infection is indeed a risk factor for persistent pulmonary vascular defects and subsequent PH development, and this could become a major public health issue in the future given the large number of individuals infected by SARS-COV-2 worldwide. Long-term studies assessing the risk of developing chronic pulmonary vascular lesions following COVID-19 infection is of great interest for both basic and clinical research and may inform on the best long-term management of survivors.

## 1 Introduction

Pulmonary hypertension (PH) is widely acknowledged as a collection of conditions that all exhibit an elevated mean pulmonary arterial pressure (>20 mm Hg) (Simonneau et al., 2019). When present, PH is associated with a worse overall prognosis. Pulmonary arterial hypertension is one class of the PH associated conditions that is also hallmarked by increased pulmonary vascular resistance and pathological remodeling. If untreated, the estimated median survival for PAH patients has been reported to be 2.8 years from the time of diagnosis (3-year survival: 48%) (D’Alonzo et al., 1991). Even with currently approved pharmacological agents, only 58%–75% of individuals with PAH may survive for 3 years (Benza et al., 2010; Humbert et al., 2010; Thenappan et al., 2010; Olsson et al., 2014). Although the precise pathobiological mechanisms of how PAH develops remains unknown. This disease may be associated with various other disorders including viral infections, such as HIV (McLaughlin et al., 2015).

Clinical evaluation and diagnostic testing in patients with coronavirus disease 2019 (COVID-19) have suggested a potential relationship between severe acute respiratory syndrome coronavirus 2 (SARS-COV-2) infection and the pathogenesis of PAH. Accumulating evidence suggests that pulmonary vascular thickening may be explicitly linked with COVID-19, implying a potential tropism of the SARS-COV-2 virus for the pulmonary vasculature (Suzuki et al., 2021). Genetic alterations that may influence the severity of COVID-19 are similar to genetic drivers of PAH (Halawa et al., 2022). The pathobiology the COVID-19-induced pulmonary vasculopathy shares many features (such as medial hypertrophy and smooth muscle cell proliferation) with that of PAH (Halawa et al., 2022). In addition, the presence of microthrombi in the pulmonary vasculature of individuals with COVID-19 during the acute phase may predispose these subjects to the development of chronic thromboembolic pulmonary hypertension (Halawa et al., 2022). These similarities raise the intriguing question of whether PAH may be a long-term sequela of SARS-COV-2 infection.

SARS-COV-2 virus is a member of a large family of viruses called coronaviruses. The primary target of SARS-COV-2 is the respiratory system, although SARS-COV-2 tropism at the cellular level is not fully defined. COVID-19 is a clinical syndrome representing the final stage of SARS-COV-2 infection and is often characterized by a proinflammatory cytokine storm leading to acute respiratory distress syndrome (ARDS) and subsequent multi-organ failure and death. The prevalence of SARS-COV-2 infection has rapidly spread across the globe, decimating the health of entire continents; and was declared a pandemic by the World Health Organization (WHO). SARS-COV-2 infection may lead to a range of cardiovascular or pulmonary complications and may be an independent risk factor for the development of pulmonary vascular diseases, including PAH, the latter of which is the subject of this review.

## 2 Observations of pulmonary vascular disease in COVID-19 patients and prevalence of PH in patients with COVID-19

### 2.1 Potential tropism of SARS-COV-2 for the pulmonary vasculature

Accumulating evidence suggests that pulmonary vascular thickening may be explicitly associated with COVID-19, implying a potential tropism of SARS-COV-2 virus for the pulmonary vasculature (Suzuki et al., 2021). Suzuki and colleagues collected post-mortem lung tissues from patients who died of COVID-19 and compared this with archived materials of lung tissue from patients who died of influenza A (H1N1) (Suzuki et al., 2021). While the pulmonary arteries of patients who died of the H1N1 influenza did not show thickened pulmonary vessels, lung histology of individuals who died of COVID-19 consistently exhibited characteristic pulmonary arterial wall thickening (Suzuki et al., 2021). In fact, the pulmonary vascular wall of COVID-19 patients was more than 2-times thicker than those of individuals who died of H1N1 influenza (Suzuki et al., 2021). The occurrence of pulmonary vascular wall thickening was also observed in the lung histology of patients who died of COVID-19 in China (Xu et al., 2020; Suzuki et al., 2021). Suzuki and colleagues demonstrated that the SARS-COV-2 spike protein may trigger cell growth signaling *via* fast activation of the mitogen-activated protein kinase (MEK)/extracellular signal-regulated kinase (ERK) pathway in human pulmonary vascular smooth muscle and endothelial cells, which raises the intriguing question of whether the spike protein predisposes subjects infected with SARS-COV-2 to develop PAH in the future, regardless of the severity of COVID-19 (Suzuki et al., 2021). Coincidentally, the human immunodeficiency virus (HIV) is the only other virus whose membrane fusion protein gp120 has been shown to trigger cell signaling that may promote pulmonary vascular remodeling, in addition to predisposing infected individuals to developing PAH (Suresh and Suzuki, 2020).

### 2.2 Genetic factors influencing COVID-19 recovery are also genetic driver of pulmonary hypertension

Marie-Pierre Dubé conducted a genetic study of symptoms duration and time to remission in 1723 outpatient individuals from COLCORONA (Dubé et al., 2021), which was a randomised clinical trial comparing the benefit of low-dose colchicine to placebo in a 40 years or older outpatient population with a recently diagnosed COVID-19 infection and with at least one high-risk criterion for severe disease (Tardif et al., 2021). The authors found a robust association with high probability of being causal between time to remission of COVID-19 symptoms and the Natriuretic Peptide Receptor 3 (Npr3) gene variants, which encodes the natriuretic peptides clearance receptor NPR-C (Dubé et al., 2021). This work provides a mechanistic understanding of the pathobiology of COVID-19 by demonstrating the importance of NPR-C signaling in promoting the remission of COVID-19 symptoms. Furthermore, these results also indicate that manipulations of NPR-C signaling pathway may have potential as a disease-modifying therapeutic target for COVID-19.

Interestingly, evidence also suggests that the Npr3 locus may have an important role in the genetics of heritable PAH (Egom et al., 2021). In addition, accumulating evidence suggests that mice lacking NPR-C develop full-blown PAH (Egom et al., 2017b). Furthermore, a specific NPR-C’s agonist, the ring-deleted atrial natriuretic peptide analogue, cANF4-23 (cANF) was shown to reduce pulmonary artery pressures in an experimental model (Egom et al., 2017a).

The above observations provide a breakthrough in the understanding of the close and potential causal relationship between the COVID-19, NPR-C and PAH. (Halawa et al., 2022) (Halawa et al., 2022) (Halawa et al., 2022)

### 2.3 Mounting evidence of SARS-COV-2-associated pulmonary hypertension throughout the course of the pandemic

In 2020, Khan and colleagues described a case of a 55-year-old female with no previously documented comorbidities who presented with exertional dyspnea, dry cough, fatigue, and episodes of syncope during exertion, only 2 months after recovery and being discharged with COVID-19 (Khan et al., 2021). The patient was subsequently diagnosed with PAH based on clinical presentation, electrocardiography, computed tomography, and transthoracic echocardiography assessment (Khan et al., 2021).

Wang and colleagues also reported a case of a 63-year-old male with no previously documented comorbidities who presented with signs and symptoms consistent with COVID-19, which was subsequently confirmed by reverse transcription polymerase chain reaction (RT-PCR). His hospital course was complicated by the development of pulmonary hypertension and acute right heart failure (Wang et al., 2020); demonstrating that the original account of PH associated with SARS-COV-2 was not an isolated phenomenon.

Around the same time, Algadeeb and colleagues reported a case of female infant who was delivered by caesarean section at 34 weeks’ gestation to a SARS-COV-2 infected, 37-year-old mother. On the fifth day of post delivery, the neonate’s investigations demonstrated a positive SARS-COV-2 infection, complicated with severe respiratory symptoms with persistent pulmonary hypertension that responded poorly to inhaled nitric oxide and respiratory support, which subsequently progressed to her death. Whether COVID-19 contributed directly to the development of pulmonary hypertension or whether the outcome was a sequela of lung pneumonia remains unclear and requires further investigation (Algadeeb et al., 2020).

Van Dongen and colleagues described a case of a 60-year-old gentleman with a past medical history significant for a prior myocardial infarction with no significant obstructive coronary disease, who presented with signs and symptoms consistent with severe COVID-19 pneumonia requiring intubation and ventilation (van Dongen et al., 2020). The patient had a computed tomography pulmonary angiogram (CTPA) revealing no signs of pulmonary embolism (PE) and thus was treated with low-molecular-weight heparin in prophylactic doses, antibiotics, methylprednisolone and tocilizumab. He had a successful recovery and was discharged 2 weeks after on 1 L/min nasal oxygen support. Unfortunately, he was re-admitted 10 days post discharge to the intensive care unit because of progressive dyspnoea and severe hypoxaemia, requiring high-flow nasal oxygen. He was subsequently diagnosed with severe pulmonary hypertension with right ventricular dysfunction by echocardiography (van Dongen et al., 2020).

In 2021, Raval and colleagues described a case of a 42-year-old female, with no previously documented comorbidities who had been recovering from COVID-19 pneumonia diagnosed 25 days prior, presenting to the emergency department with shortness of breath. The initial investigations performed on admission revealed normal echocardiography, elevated pro B-type natriuretic peptide and a positive COVID-19 RT-PCR and antibody test. During her hospital stay she underwent CTPA revealing no PE. A nuclear medicine lung ventilation-perfusion was normal and repeated echocardiography showed PAH, which was later confirmed invasively with right heart catheterization. The patient was discharged with the diagnosis of COVID-19 associated pulmonary artery hypertension and was started on the phosphodiesterase-5 inhibitor, tadalafil (Raval et al., 2021).

Maria Vlachou and colleagues reported a case of a 53-year-old lady with no previously documented comorbidities who presented with signs and symptoms consistent with COVID-19. On day seven, the patient complained of non-pleuritic chest pain and increased breathlessness; an electrocardiogram revealed new widespread T wave inversion and cardiac biomarkers rose from 16 to 38 ng/L. A computed tomography pulmonary angiography or CTPA was performed and showed no evidence of PE, however right ventricular dilatation was evident. She was treated as non-ST segment elevation myocardial infarction with a percutaneous coronary intervention. In view of her right ventricular dilatation, right heart catheterization was performed and revealed a mean pulmonary arterial pressure of 36 mmHg, with a pulmonary capillary wedge pressure of 11 mmHg, a cardiac index of 1.6 L/min/m^2^, and a pulmonary vascular resistance of 10 Wood units (Vlachou et al., 2021).

Salcin and colleagues reported a case of a 62-year-old female with a past medical history significant for previous COVID-19 infection, hypothyroidism, and hypertension, who presented again with signs and symptoms consistent with COVID-19-related illness (Salcin and Fontem, 2021). Her clinical course was complicated with an episode of acute respiratory distress syndrome requiring an intensive care unit admission and she was twice intubated. During her 6 weeks post-discharge follow up, the patient reported dyspnea, hypoxia, new peripheral edema and continued oxygen dependence. She subsequently underwent right heart catheterization for further evaluation and was found to have newly developed PAH (Salcin and Fontem, 2021).

Karmakar and colleagues reported a case of a 60-year-old male with a past medical history significant for elevated BMI and pre-diabetes, who presented again with signs and symptoms consistent with COVID-19-related illness. After a long hospital stay and a complicated clinical course requiring an intensive care unit admission, he was discharged home with intermittent oxygen therapy. Three months after recovering from active COVID-19, he underwent further investigations revealing new (not previously documented) extensive pulmonary fibrosis and pulmonary hypertension (Karmakar et al., 2021).

Also in 2022, Cueto-Robledo and colleagues reported a case of a 58-year-old female with a past medical history significant for hypertension, who presented with progressively worsening dyspnea on exertion 3 months post severe COVID-19 pneumonia (Cueto-Robledo et al., 2022). She subsequently underwent right heart catheterization for further evaluation and was found to have newly developed PAH (Cueto-Robledo et al., 2022).

Therefore, in the early 2020s, the possibility of an association between SARS-COV-2 infection and the development of pulmonary vascular diseases, and consequently PAH, was established. These early observations suggest the following epidemiology, clinical and pathological characteristics for SARS-COV-2-associated PH:

1. SARS-COV-2-associated PH may be more prevalent in men, which contrasts with the high female predominance observed in idiopathic PAH.
2. PH may occur in early and late stages of SARS-COV-2 infection.
3. The severity of COVID-19 may not affect the risk of developing PH (which may occur regardless of the severity including in asymptomatic individuals with SARS-COV-2 infection), however it may influence the timing of PH’s development (early and acutely in severe disease) (Haberecker et al., 2022)
4. PH may contribute to rapid clinical deterioration in SARS-COV-2.
5. SARS-COV-2-associated PH may be responsive to conventional PAH-related therapies.
6. SARS-COV-2-associated thromboembolic PH may not be responsive to anticoagulation therapy (Borczuk et al., 2020)
7. The estimated prevalence of PH in a non-ICU and ICU patients with SARS-COV-2 is up to 12% (Pagnesi et al., 2020) and up to 39% respectively (Norderfeldt et al., 2021).
8. The pathology of PH in SARS-COV-2 may overlap with PH

## 3 Pathobiology of pulmonary vascular diseases in SARS-COV-2

### 3.1 SARS-COV-2 infection and vascular thrombosis

#### 3.1.1 Microvascular thrombosis

Ackermann and colleagues described autopsy findings in seven individuals who died from SARS-COV-2 infection and compared them with those of subjects who died from H1N1 infection-induced acute respiratory distress syndrome and 10 age-matched, uninfected control lungs. The pulmonary autopsy findings of patients with SARS-COV-2 infection exhibited distinctive vascular characteristics, including severe endothelial damage with evidence of intracellular SARS-COV-2 virus and disruption of the plasma *membranes* of vascular *endothelial* cells (Ackermann et al., 2020b). The pulmonary vessels autopsy findings of patients with COVID-19 also exhibited widespread thrombosis with microangiopathy and alveolar capillary occlusion (Ackermann et al., 2020b). Pulmonary alveolar capillary microthrombi were 9 times as prevalent in individuals with SARS-COV-2 infection as in subjects with H1N1 (Ackermann et al., 2020b). Similarly, pulmonary intussusceptive angiogenesis was 2.7 times as prevalent in individuals with SARS-COV-2 infection as in subjects with H1N1 (Ackermann et al., 2020b).

Borczuk and colleagues describe pulmonary autopsy findings in a series of 68 cases of SARS-COV-2 infection (Borczuk et al., 2020). The authors reported thrombi and vascular injury of small- and mid-sized pulmonary vessels in 84% of individuals with SARS-COV-2 infection (Borczuk et al., 2020). These pulmonary vascular thrombi were sometimes not associated with areas of acute lung injury (Borczuk et al., 2020). Interestingly, the frequency of pulmonary vascular macrothrombi and microthrombi was independent of anticoagulation therapy (Borczuk et al., 2020).

Furthermore, Haberecker and colleagues retrospectively assessed the relationship between autopsy-based cause of death, magnitude of pulmonary vascular injury and clinical parameters in 15 COVID-19 autopsies with a different clinical course. Although severe pulmonary endotheliitis correlated with COVID-19 associated death, clinical disease severity parameters did not correlate with COVID-19 associated death nor did they influence the occurrence of pulmonary vascular thrombosis/microthrombi (Haberecker et al., 2022).

#### 3.1.2 Mismatched perfusion defects as a consequence of microvascular thrombosis

Several studies have reported mismatched perfusion defects in early–post-COVID-19 patients, which may reflect a thromboembolic process (Klok et al., 2020; Mueller-Peltzer et al., 2020; Roncon et al., 2020; Idilman et al., 2021; Mandal et al., 2021; Sajal et al., 2021). As hinted above, widespread microthrombi have been reported in multiple organ systems in patients with COVID-19. Wichmann and colleagues demonstrated that approximately 60% of patients with COVID-19 exhibited deep venous thrombosis, which was not suspected before death (Wichmann et al., 2020). As mentioned above, Ackermann and colleagues have also demonstrated that approximately 60% of patients with COVID-19 exhibit widespread thrombosis with severe endothelial injury (Ackermann et al., 2020b).

The principal constituents of microthrombi tend to be transient: red blood cells, which usually have 2–4-month lifespan, and fibrin, which is degraded by plasmin into fibrin degradation products. D-dimer is the principal fibrin degradation products, which has been used as a predictive biomarker of fibrin formation and breakdown. Abnormal plasma D-dimer levels have been reported in patients with COVID-19 and has been associated with increased morbidity and mortality (Berger et al., 2020; Poudel et al., 2021; Zhan et al., 2021). In a study conducted by Berger et colleagues (Berger et al., 2020), 76% of hospitalized patients with COVID-19 presented with an elevated D-dimer and 86% of them had an elevated D-dimer at some point during the course of hospitalization (Berger et al., 2020). Lehmann and colleagues investigated the rate of persistent elevation of D-dimer and its association with thromboembolic complications in patients who recovered from COVID-19 (Lehmann et al., 2021). The authors found persistent D-dimer elevation in 15% of patients at 3 months follow up after their initial bout of COVID-19. Interestingly, patients with persistent D-dimer elevation still exhibited a significant lower mean partial pressure of oxygen and increased alveolar-to-arterial oxygen gradient, reflecting a persistent ventilation/perfusion (V/Q) mismatch, a vital component in the pathophysiology of hypoxemia (Lehmann et al., 2021). Persistent D-dimer elevation was also reported in more than 25% of patients up to 4 months post-SARS-COV-2 infection (Townsend et al., 2021), which exceeds the life span of erythrocytes and fibrin. The residual ventilation/perfusion (V/Q) mismatch or perfusion defects may reflect microthrombi that have been remodeled into chronic fibrotic tissue (Morris et al., 2006).

#### 3.1.3 Mechanisms of the resolution or persistence of mismatched perfusion defects

The mechanisms through which the microemboli in some COVID-19 patients evade normal plasmin-mediated lysis leading to incomplete resolution of the thrombus and thus persistent perfusion defects remain unknown. This explains, at least partly, the current inability to predict which patients with a history of COVID-19 illness will go on to develop PH/chronic thromboembolic pulmonary hypertension (CTEPH). Several pathobiological processes may account for incomplete microthrombi resolution.

Morris and colleagues demonstrated that fibrin clots from subjects with CTEPH may be resistant to plasmin-mediated lysis, when compared with fibrin clots from healthy control individuals (Morris et al., 2006). The relative increased resistance to endogenous thrombolysis may be multifactorial, including, but not limited to, alterations in fibrin structure such as persistence of the N-terminus of the β-chain, as well as polymorphisms affecting the fibrinogen α–α chain cross-linkage (Morris et al., 2006; Suntharalingam et al., 2008).

**FIGURE 1:**
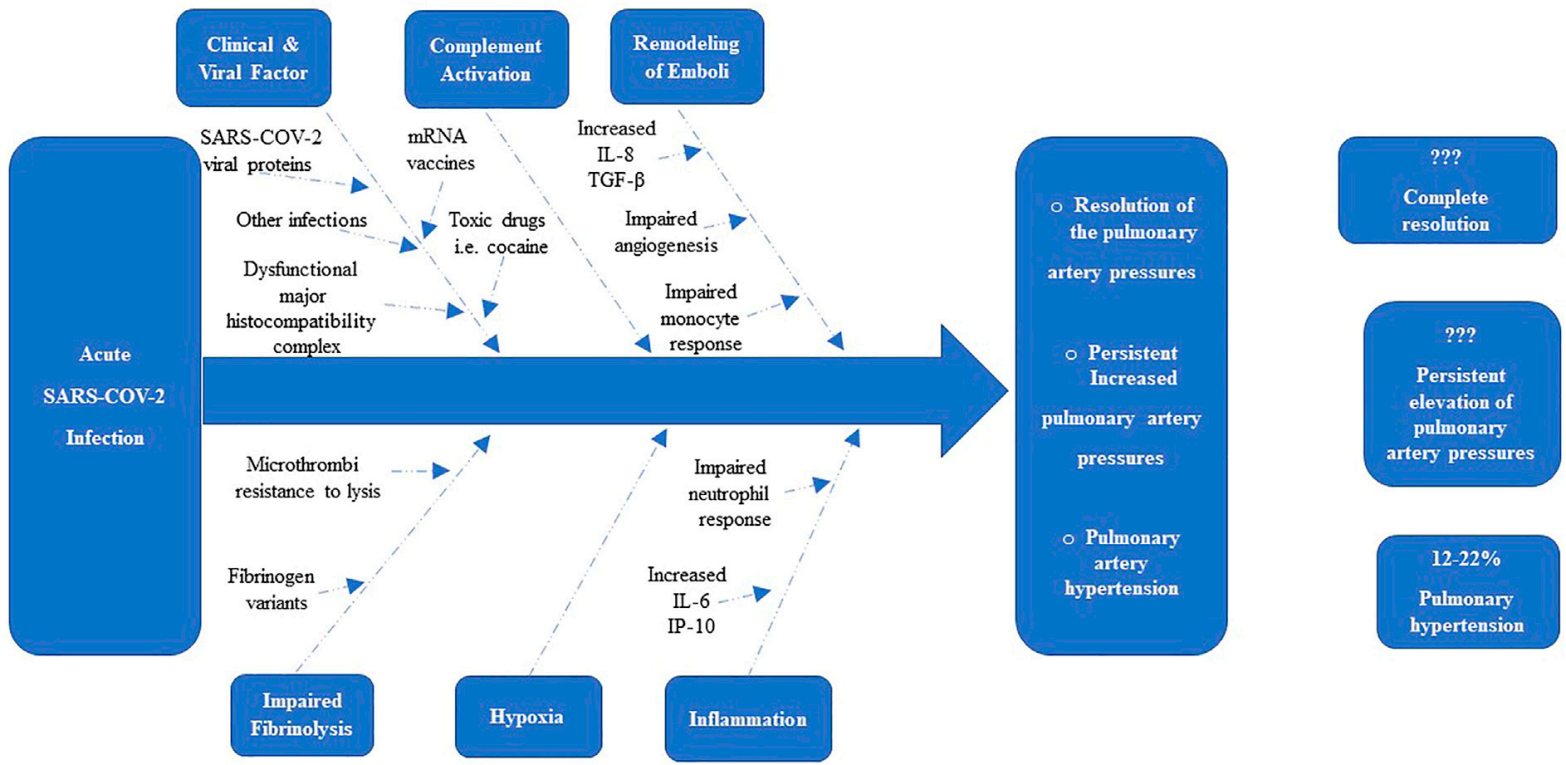
Determinants of the resolution or persistence of Elevation of Pulmonary Pressures after acute SARS-COV-2 Infection (Morris et al., 2006).

Evidence suggests that the N-terminus of the β-chain of fibrin may have angiogenic activity, and its persistence within the pulmonary vasculature may act as a stimulus for the proliferation of endothelial cells and fibroblasts into the clot residua and thereby promoting the formation of capillary-like structures, as well as the transition from acute microthrombi into organized fibrotic tissue (Thompson et al., 1992; Martinez et al., 2001; Morris et al., 2006). Other factors present in the unique microenvironment created by the unresolved microthrombi may trigger migration of resident adventitial fibroblasts, dysregulated differentiation and proliferation of the mesenchymal progenitor cells into a myofibroblast cell phenotype, as well as activation of matrix and collagen production (Yi et al., 2000; Firth et al., 2010; Zabini et al., 2012). These factors, specifically thrombin, may also initiate a stimulus/coupling response leading to dysfunctional endothelial cells (Garcia et al., 1993; Sakao et al., 2011; Zabini et al., 2012). The functional consequence of these cellular changes may contribute, at least in part, to pulmonary vascular remodeling that may be observed in these patients (Yi et al., 2000; Firth et al., 2010; Zabini et al., 2012), The potential determinants of the resolution or persistence of microemboli after an acute SARS-COV-2 infection are summarised in Figure 1.

### 3.2 SARS-COV-2-related factors that mediate pulmonary vascular remodeling

#### 3.2.1 SARS-COV-2 spike protein

Evidence suggests that the SARS-COV-2 spike protein may begin the intricate pathogenesis and pulmonary vascular remodeling pattern of SARS-COV-2-associated PH (Suzuki et al., 2021). The SARS-COV-2 spike protein is encoded by the SARS-COV-2 gene, is exposed on the surface of the SARS-COV-2 virus and facilitates entry of SARS-COV-2 into the host target cell *via* membrane fusion (Shang et al., 2020; Tai et al., 2020; Walls et al., 2020; Yan et al., 2020; Suzuki et al., 2021). In fact, the SARS-COV-2 spike protein is comprised of two subunits: the S1 subunit, which contains the ACE2 receptor-binding domain and the S2 subunit, which is responsible for the fusion between cell and viral membranes for cell entry (Suzuki et al., 2021). This fusion initiates a cascade of conformational changes that leads to fusion of the SARS-COV-2 virus with the host cell membrane with subsequent intracellular entry of the SARS-COV-2 virions (Jackson et al., 2022). Evidence suggests that even transient exposure of picomolar concentrations of S1 protein alone without the rest of the viral components is sufficient to trigger cell growth signaling *via* a fast activation of MEK/ERK pathway in human pulmonary vascular smooth muscle and endothelial cells (Suzuki et al., 2021). This may promote hyperplasia and/or hypertrophy of the pulmonary vasculature in humans as MEK phosphorylation has been associated with de-differentiation, nuclear activation and proliferation of pulmonary vascular smooth muscle cells in pulmonary hypertension (Xing et al., 2019; Suzuki et al., 2021).

#### 3.2.2 Immune system dysregulation

Dysregulation of the immune and inflammatory systems have been reported as a driver of the critical course of SARS-COV-2 infection (Kosmaczewska and Frydecka, 2020). Kennedy and colleagues found that individuals with mild or asymptomatic SARS-COV-2 infections may have sustained immune activation and systemic inflammation that persists for at least 3 months after their initial illness (Kennedy et al., 2021). Phetsouphanh and colleagues studied a cohort of long COVID patients compared to age- and gender-matched recovered individuals without long COVID, unexposed donors, and patients infected with other coronaviruses; and found that individuals with long COVID have prolonged immunological and inflammatory dysfunction even 8 months following their initial mild-to-moderate SARS-COV-2 infection (Phetsouphanh et al., 2022). This is relevant as evidence suggests that chronic immune activation and inflammation may contribute to the development of pulmonary vascular diseases. Evidence also suggests that chronic immune activation may promote the development of PAH in this model (Kling et al., 2018).

### 3.3 Other factors contributing to SARS-COV-2-Associated Pulmonary Hypertension

#### 3.3.1 Stimulant use

COVID-19 patients with a history of “stimulant” use such as amphetamine, methamphetamine, and cocaine may be 7 to 10 times more likely to develop SARS-COV-2-associated PH as these drugs may act as disease modifiers and promote pulmonary vascular remodeling (Chin et al., 2006). Wang and colleagues conducted a retrospective case-control study of electronic health records data of patients with substance use disorders, particular opioid use disorders, and found that these individuals were at significant increased risk for COVID-19 and its adverse outcomes including severe disease requiring hospitalization or resulting in death (Wang et al., 2021). Similarly, Lindsey Wang and colleagues also performed a population-based cohort study to investigate the risk and outcomes of COVID-19 breakthrough infection in fully vaccinated, substance use disorders patients and found higher risks for breakthrough infection compared with matched non-substance use disorders cohorts, with the highest risks for those with a history of cocaine or cannabis use (Wang et al., 2022).

#### 3.3.2 Co-infection with HIV or schistosoma

**FIGURE 2:**
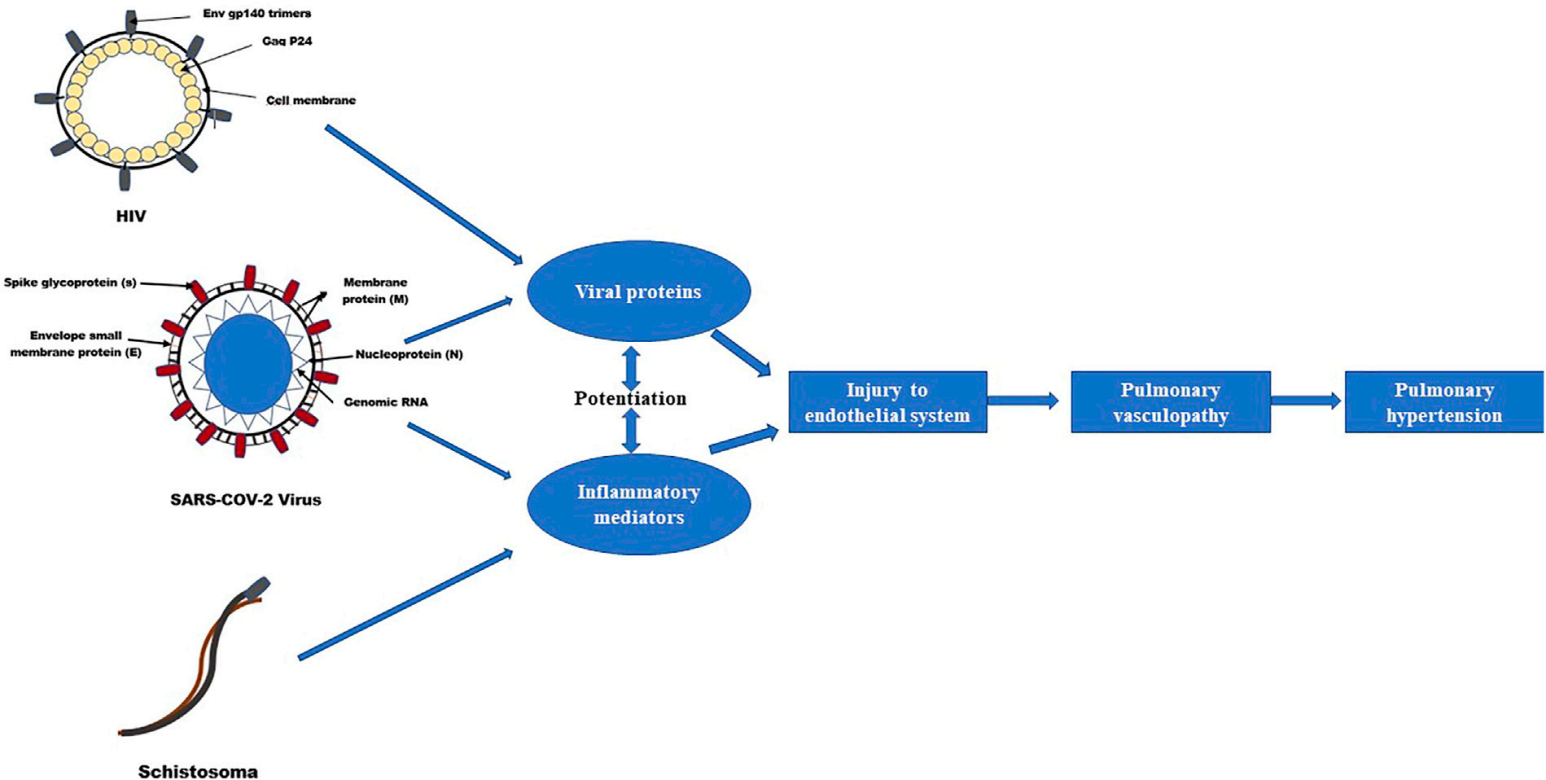
Effects of co-exposure of SARS-COV-2, HIV and Schistosomiasis on pulmonary vascular disease development (Butrous, 2015).

Co-morbid infection with certain pathogens may also be a supplementary contributory factor to the development of SARS-COV-2-associated PH (as illustrated in Figure 2). Schistosoma and the HIV infections are independent risk factors for the development of pulmonary vascular diseases, including PAH (Kolosionek et al., 2013; Butrous, 2015). Although the effects of co-infection with SARS-COV-2 on pulmonary vascular disease development are still unknown; co-exposure of these pathogens may potentiate pulmonary vascular disease resulting in different remodeling phenotypes as the dysregulation of inflammatory responses induced by one may influence the pattern of pulmonary vascular remodeling mediated by the other (Morales Cano et al., 2019).

As hinted above, the evidence suggests that chronic immune activation and inflammation may contribute to the development of pulmonary vascular diseases. Patients with autoimmune disease or chronic inflammatory disease may, therefore, also be at increased risk of severe SARS-COV-2 mediated PH due to functional impairment of the immune system and persistent inflammation (Kanwugu and Adadi, 2021).

## 4 The role of SARS-COV-2 in the pathogenesis of pulmonary vascular diseases: Contemporary hypotheses

### 4.1 Endothelial injury and vascular remodelling

**FIGURE 3:**
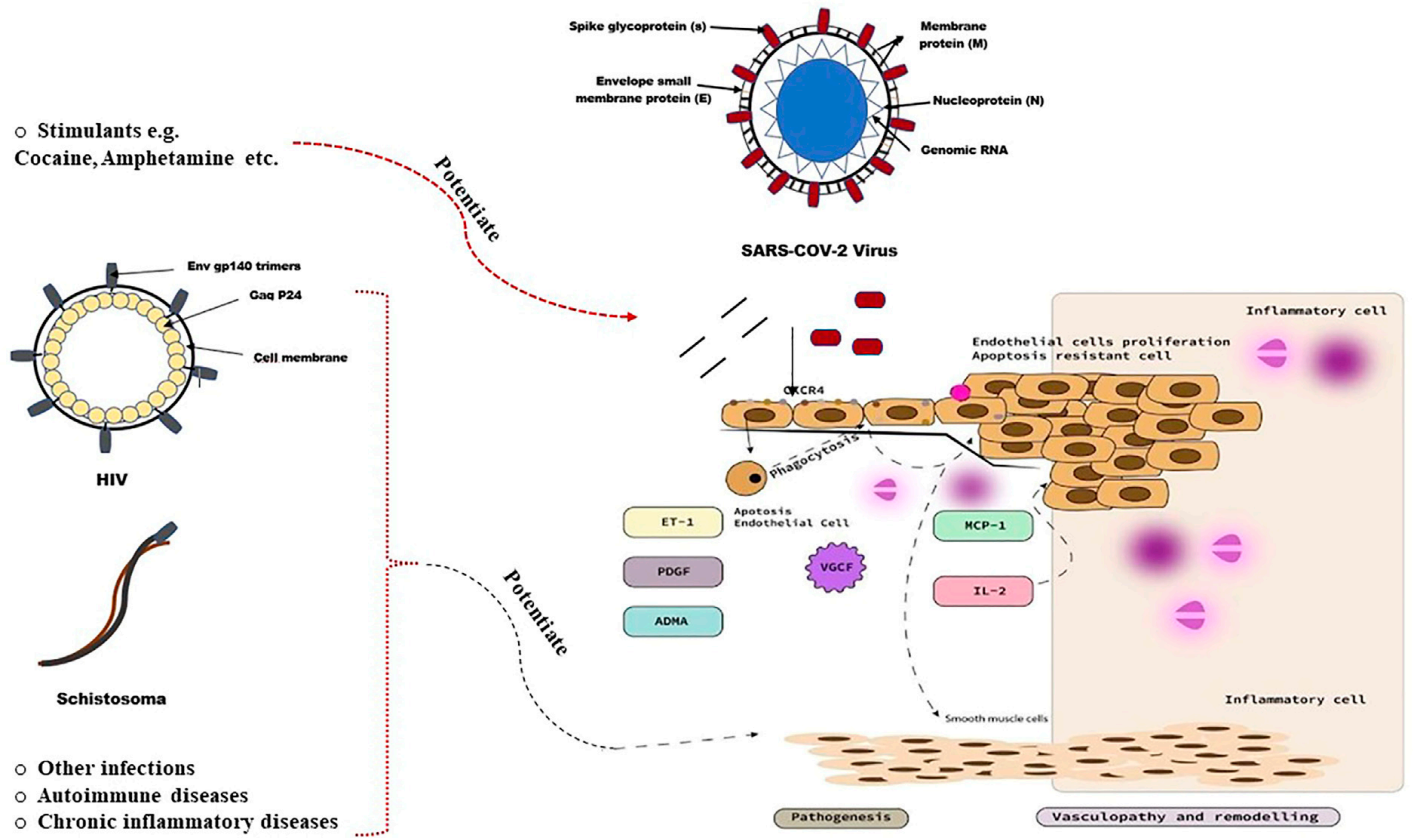
Pathobiology of pulmonary vascular disease in SARS-COV-2 infected individuals (Butrous, 2015).

The exact underlying pathobiology of pulmonary vascular disease in SARS-COV-2 infected individuals remains uncertain. However, the current framework explaining the pathobiology of SARS-COV-2-associated PH based on the aforementioned observations and the empirical evidence is summarised in Figure 3. Pulmonary vascular diseases are often characterised by excessive pulmonary vasoconstriction and remodeling processes of the pulmonary vasculature, particularly the distal pulmonary vessels. The pathobiological process classically starts with injury to the pulmonary endothelial cells (Humbert et al., 2019). It remains to be determined whether theSARS-COV-2 virus directly infects endothelial cells, causing endothelial cell injury and initiating pulmonary vascular remodeling. In fact, the exact route of entry of SARS-COV-2 into the pulmonary vascular wall is not well understood. Although some studies suggest that pulmonary vascular endothelial cells may represent a primary target of the SARS-COV-2 virus (Delorey et al., 2021), the lack of detectable expression of two important receptors (angiotensin-converting enzyme 2 (ACE2) and Transmembrane Serine Protease 2 (TMPRSS2) (McCracken et al., 2021), which are essential for the entry of SARS-COV-2 into cells, indicates that SARS-COV-2-induced pulmonary vascular endotheliitis may be caused by indirect perivascular inflammation and complement activation rather than by direct viral infection of pulmonary vascular endothelial cells (Halawa et al., 2022). An alternative route of entry of SARS-COV-2 into the pulmonary vascular wall may occur *via* the pulmonary vascular smooth muscle cells and/or fibroblasts (Halawa et al., 2022). The pulmonary vascular smooth muscle cells, as well as lung fibroblasts, express the two important receptors ACE2 and TMPRSS2 indicating that the observed alterations of these cells in patients with COVID-19 may be the result of a direct or an indirect SARS-COV-2 infection (Hamming et al., 2004; Muus et al., 2021; Halawa et al., 2022).

### 4.2 The major mechanisms mediating injury

Multiple mechanisms may be implicated in SARS-COV-2 mediated pulmonary vascular injury including, but not limited to, hypoxia, inflammation, and complement.

#### 4.2.1 Hypoxia

Evidence suggests that 20%–40% of patients with COVID-19 may be hypoxic despite an apparent lack of dyspnoea (silent hypoxia) (Tobin et al., 2020; Swenson et al., 2021). Hypoxia is known to cause pulmonary vascular endothelial cell injury (74,75) *via* multiple mechanisms including activation of the NF-κB transcription factor (Taylor and Colgan, 2017; Marchetti, 2020), promotion of a cytokine storm (Jahani et al., 2020) and complement activation (Ward et al., 2016). In addition, hypoxia may trigger the overexpression of ACE2 receptors, thereby increasing the risk of SARS-COV-2 associated pulmonary vascular injury (Zhang et al., 2009).

#### 4.2.2 Inflammation

Several inflammatory signalling pathways have been reported to be dysregulated in patients with COVID-19 (Halawa et al., 2022). The potential underlying pathobiological mechanisms may include SARS-COV-2 associated pulmonary vascular endothelial injury (Ackermann et al., 2020b), which subsequently promotes leukocyte infiltration and alteration of the coagulation control, leading to a pro-coagulant environment in the vasculature; SARS-COV-2-induced cytokine storm (Levi and Coppens, 2021) as well as a SARS-COV-2-mediated type 3 hypersensitivity reaction (Roncati et al., 2020).

#### 4.2.3 Complement

Evidence suggests that SARS-COV-2 infection may trigger an uncontrolled activation of the complement system, leading to complement deposition in the pulmonary microvasculature as well as excessive release of inflammatory cytokines (Carvelli et al., 2020; Noris et al., 2020; Stenmark et al., 2021). This deposition may in turn promote the activation of the membrane attack complex, resulting in microvascular endothelial damage and activation of the coagulation pathways (Magro et al., 2020; Halawa et al., 2022).

### 4.3 SARS-COV-2 viral proteins and inflammatory chemokines

As alluded to above, evidence suggests that SARS-COV-2 viral proteins may also contribute to endothelial cell injury and initiate pulmonary vascular remodeling. In fact, the S1 protein may promote cell growth signaling *via* fast activation of MEK/ERK pathway in human pulmonary vascular smooth muscle and endothelial cells (Suzuki et al., 2021). This may, in turn, stimulate maladaptive pulmonary vascular cell growth patterns, including de-differentiation, proliferation, endothelial cell apoptosis, nuclear activation and pulmonary vascular smooth muscle cell apoptosis resistance, similar to the defining characteristics of non-SARS-COV-2 forms of PH (Xing et al., 2019; Potus et al., 2020; Suzuki et al., 2021).

SARS-COV-2 membrane glycoprotein M with the assistance of the nucleocapsid protein N may induce caspase-mediated signaling pathways apoptosis *via* interacting with PDK1 and inhibiting the activation of PDK1-PKB/Akt signaling (Ren et al., 2021). Interestingly, the SARS-COV-2 spike protein is reported to increase intracellular reactive oxygen species (ROS) levels, which, in turn, may trigger autophagic and inflammatory responses, as well as programmed cell death or apoptosis in infected pulmonary vascular endothelial cells (Li et al., 2021). Additionally, SARS-COV-2 viral proteins may also induce sustained elevation of the potent vasoconstrictor endothelin-1 in pulmonary vascular endothelial cells (Abraham et al., 2022; Willems et al., 2022). Apoptotic cell clearance by professional phagocytes (by phagocytosis), performed as part of the long-term maintenance of tissue homeostasis, may in fact trigger the release of further cytokines and growth factors, which, collectively, may promote excessive and complex pulmonary vascular cell proliferation and remodeling, and PH (Rafikova et al., 2019).

SARS-COV-2 viral proteins may also interact with different other types of cell surface receptors to increase local concentrations of specific inflammatory cytokines known to mediate pulmonary vascular remodeling such as platelet-derived growth factors (PDGFs), vascular endothelial growth factor (VEGF), interferon gamma inducible protein 10 kD (IP-10) and fibroblast growth factor-2 (FGF2) (Pang et al., 2021; Petrey et al., 2021).

Circulating chemokines may also promote SARS-COV-2-associated PH *via* an interaction with pulmonary vascular endothelial cell receptors. One such chemokine is CXCR4, which was found to be highly upregulated in the lungs of patients with COVID-19 (Ackermann et al., 2020a; Khalil et al., 2021). In addition, SARS-COV-2 viral proteins may interact with different types of cell surface receptors of pulmonary vasculature to induce upregulation of the p38 MAPK pathway (Grimes and Grimes, 2020), VCAM-1 expression (Birnhuber et al., 2021), nuclear factor–kappa B (NF-κB) translocation *via* the reduction of the dual-specificity phosphatases (Goel et al., 2021), and up-regulation of the hypoxia-inducible factor–1α (HIF-1) (Taniguchi-Ponciano et al., 2021), which, collectively, are implicated in the pathobiology of pulmonary vascular remodeling and dysfunction (Butrous, 2020).

SARS-COV-2-mediated release of cytokines such as IL-6, or increased levels of the endogenous competitive inhibitor of endothelial nitric oxide synthase, asymmetrical dimethylarginine (ADMA), may also promotes endothelial dysfunction and vascular smooth muscle cell proliferation and, thus, contribute to SARS-COV-2-associated PH (Hannemann et al., 2021; Jones and Hunter, 2021).

In addition, SARS-COV-2 viral proteins may modulate monocyte chemotactic protein-1 (MCP-1) and the release of IL-2, which may promote pulmonary vascular remodeling (Shi et al., 2020; Xi et al., 2021; Kundu et al., 2022), particularly in HIV as well as in Schistosomiasis infections (Lehmann et al., 2019; de Oliveira Nóbrega et al., 2021; Silva et al., 2021) (Figure 2). It remains to be determined whether the overlap in cytokines profiling patterns in SARS-COV-2, HIV and Schistosomiasis promotes synergistic PH development in coinfected patients.

### 4.4 Synergistic effect of “stimulants”

The adverse effects of SARS-COV-2 viral proteins on the pulmonary vascular endothelium can also be potentiated by the use of “stimulants” (Chin et al., 2006). Some “stimulant” use, such as cocaine, may induce a synergistic effect with SARS-COV-2 viral proteins to increase the expression of host-encoded microRNA (miRNA). This can potentially modulate bone morphogenetic protein receptor expression in the pulmonary vascular smooth muscles cells, such as miR-423-5p, miR-23a-3p and miR-195-5p, to trigger pulmonary vascular remodeling (Dalvi et al., 2018; Farr et al., 2021).

### 4.5 Dysfunctional major histocompatibility complexes

Evidence suggests that the development of PAH in the setting of SARS-COV-2 infection may also occur as a result of dysfunctional major histocompatibility complex (MHC), indicating an autoimmune basis for the disease (Figure 3). This is evidenced by the finding that the viral protein encoded by open reading frame 8 (ORF8) of SARS-COV-2 may downregulate the gene encoding MHC class I (Zhang et al., 2021). Ji-Seung Yoo and colleagues demonstrated that in COVID-19 patients, SARS-COV-2 ORF6 protein suppresses the MHC class I transactivator NOD-like receptor family CARD domain containing five, both transcriptionally and functionally (Yoo et al., 2021). These observations suggest that SARS-COV-2-associated PH may reflect a host response to SARS-COV-2, determined by a set of alleles located within MHC. Similarly, the role of MHC abnormalities in HIV-associated pulmonary vascular diseases has also been documented (Morse et al., 1996).

## 5 The role of the “second hit” in the pathogenesis of SARS-COV-2 associated pulmonary hypertension

**FIGURE 4:**
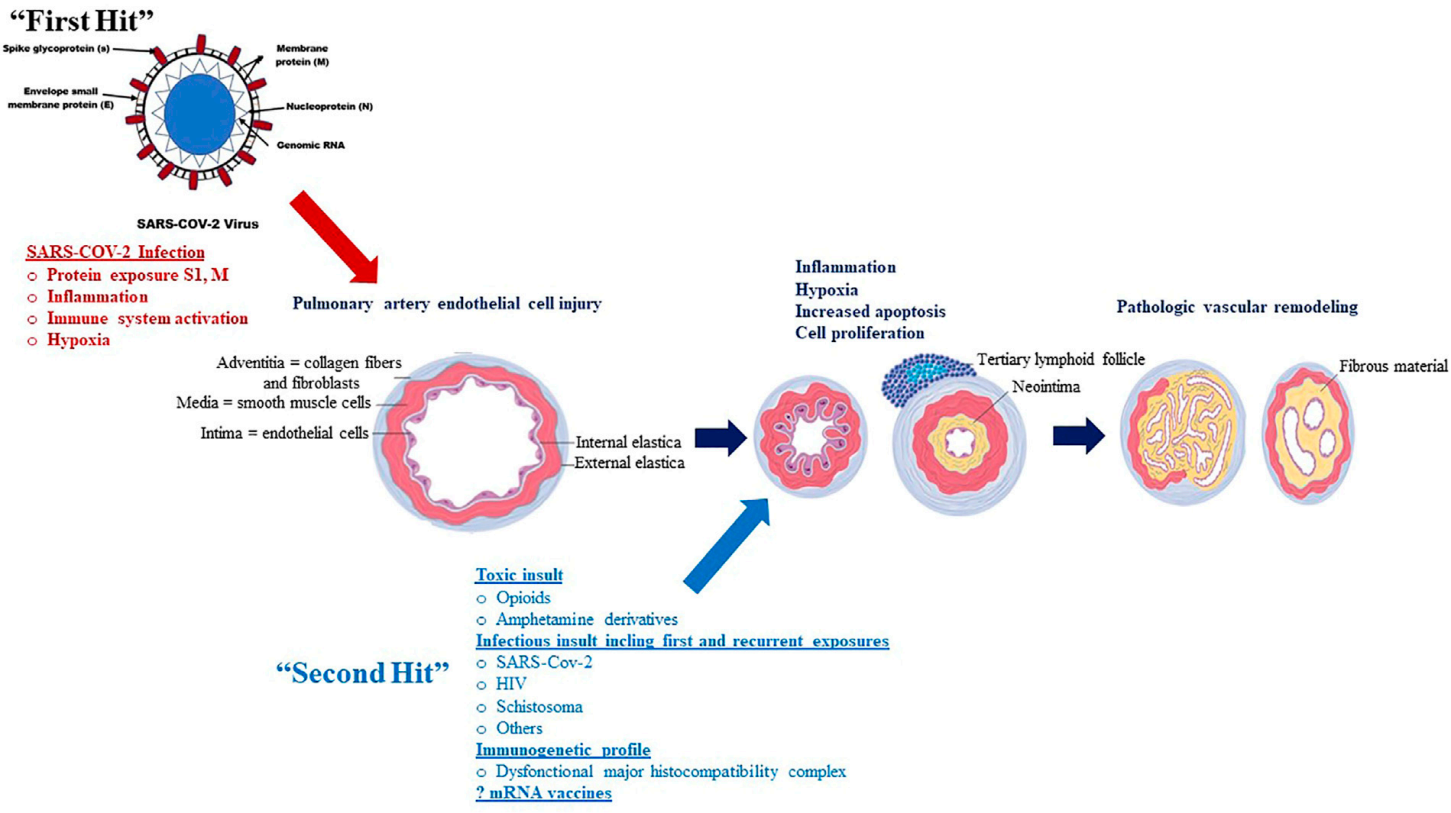
The potential association between SARS-COV-2 infection and PAH: A “Multiple Hit” Hypothesis (McLaughlin et al., 2015).

The aforementioned associations between SARS-COV-2 infection and PH intuitively leads to the following questions. Firstly, what role does severity of SARS-COV-2 infection or COVID-19 play in PH pathogenesis? Secondly, can sequential insults (such as recurrent exposure to SARS-COV-2 infection), which may individually be innocuous/asymptomatic or causing mild disease, lead to overwhelming physiologic reactions (called “Second Hit”) (as illustrated in Figure 4).

Although SARS-COV-2 viral proteins display a likely pivotal role in the pathogenesis of SARS-COV-2 associated PH, not all patients with COVID-19 develop the disease, leading to the investigation of other risk factors, or “second-hit” events as illustrated in Figure 1.

Individuals with recurrent exposure to SARS-COV-2 (asymptomatic or mild COVID-19) may represent one potential “second hit” that may contribute to the development of SARS-COV-2 associated PH. Toxic drugs such as opioids or amphetamine derivatives may also be another potential “second hit” that may contribute to the development of SARS-COV-2 associated PH; SARS-COV-2 viral proteins and opioid use may interact to increase apoptosis, with endothelial injury subsequently promoting the proliferation of apoptosis-resistant cells, which in turn may lead to angio-obliteration *via* increased production of reactive oxygen species (Spikes et al., 2012; Ataei et al., 2020). Other toxic agents such as cocaine or amphetamine derivatives may also increase risk of SARS-COV-2 associated PH through increase in pulmonary artery endothelial cell permeability and smooth muscle cell proliferation (Dhillon et al., 2011; Wang et al., 2021). These hypothetical mechanisms, however, by which these drugs contribute to pulmonary vasculopathy in SARS-COV-2-PAH, remain to be demonstrated.

These hypothetical observations support the concept that SARS-COV-2 associated PH may be a “multiple hit” phenomenon and that recurrent asymptomatic or mild COVID-19, as well as toxic drug-induced endothelial cell injury, may represent examples of such insults. Identifying these risk factors in addition to understanding the multiple hits in the pathogenesis of SARS-COV-2 associated PH will help identify high-risk individuals and optimize medical management for this deadly disease.

## 6 The global impact of SARS-COV-2-associated pulmonary hypertension

To date, over 500 million individuals have been infected with SARS-COV-2 virus worldwide, with a case fatality rate of 2%3% (Cao et al., 2020; World Health Organization, 2022). As more people may have been exposed to SARS-COV-2 virus without the official diagnosis of COVID-19, the true number of people who have recovered from the SARS-COV-2 infection is expected to be substantial. Given the large number of individuals being infected with SARS-COV-2 during the pandemic and that most subjects recover from severe, mild or asymptomatic COVID-19, it is imperative to understand how the health of recovered subjects may be affected long-term. PH is one potential lethal consequence that has been considered and needs to be monitored.

Early estimates suggest that the prevalence of PH is ≈ 22% in patients with SARS-COV-2 infection. This estimate is supported by data from a systematic review and meta-analysis investigating the prevalence of right ventricular dysfunction and pulmonary hypertension in COVID-19 cases, even in the non-advanced stage. A total of 16 eligible studies (1,728 patients) were included. Most of the studies defined PH as pulmonary artery systolic pressure greater than 35–40 mmHg. The authors found that the prevalence of pulmonary hypertension and right ventricular dysfunction in COVID-19 was 22% and 19%, respectively and these were associated with increased hospital morbidity and mortality (Oktaviono et al., 2021).

However, Pagnesi and colleagues studied 211 hospitalised, non-intensive care unit (ICU) patients with moderate to severe COVID-19 but without critical pulmonary involvement (Pagnesi et al., 2020) and found that the prevalence of SARS-COV-2-related PH was as high as 12%.

Rossi and colleagues performed a right heart catheterization (RHC) and a 6-min walking test (6MWT) in 25 consecutive COVID-19 survivors who presented to their divisional outpatient clinic with signs and symptoms suggestive of “Long COVID” (Rossi et al., 2022). The authors compared these RHC and 6MWT data with those of their 25-counterpart gender- and age-matched asymptomatic COVID-19 survivors. Interestingly, all patients with “Long COVID” were found to have pulmonary arterial hypertension and severe right ventricular systolic dysfunction (Rossi et al., 2022).

Norderfeldt and colleagues investigated the occurrence of acute pulmonary hypertension in severe COVID-19 patients admitted in the intensive care unit (Norderfeldt et al., 2021). The authors found that 39% of patient with severe COVID-19 had acute pulmonary hypertension, which is much higher than was reported by other groups (Norderfeldt et al., 2021). This study likely included mainly acute-, severe- or critically-ill subjects. However, individuals with known pre-existing chronic pulmonary hypertension and a complex pre-existing comorbidity, that would have rendered trans-thoracic echocardiography interpretation difficult, were excluded.

Tudoran and colleagues investigated the presence and severity of PH in a group of 91 patients, aged between 30 and 55 years without previous significant cardiovascular or pulmonary diseases that could explain the existence of PH, at 2 months after they recovered from a mild/moderate COVID-19 related pulmonary infection (Tudoran et al., 2021). Most of these patients had a baseline normal transthoracic echocardiography and all of them were treated prophylactically with either apixaban or rivaroxaban from their admission up to 40 days post discharge. At 2 months post discharge, the patients underwent a transthoracic echocardiography and approximately 20% of them had an abnormally elevated pulmonary artery systolic pressure with 6%–7% already showing evidence of pulmonary vascular disease and 10%–11% experiencing signs of right ventricular dysfunction. This is striking considering the young age of this cohort (all under 55 years) and the short time course of PH development despite the widespread use of oral anticoagulants (to reduce the potential impact of the thromboembolic processes). The authors also investigated whether there was a correlation between the severity of PH, the extent of the initial lung injury, and the alterations of inflammatory markers (Tudoran et al., 2021). There were statistically significant correlations between pulmonary artery systolic pressures, the initial severity of lung injury, the number of days patients remain PCR positive for SARS-COV-2, as well as with c-reactive protein and interleukin-6 levels, with the highest observed correlations between pulmonary artery systolic pressure levels and the inflammatory markers (Tudoran et al., 2021). They authors performed a multivariate linear regression analysis and after adjustment for potential confounders, the initial severity of lung injury, D-dimers, fibrinogen, interleukin-6, and hospital O2 saturation were the most significant predictor factors for the development of PH and right ventricular dysfunction.

The observed differences in the prevalence of PH between studies are likely related to the heterogeneity across the methods for diagnosis of PH.

It is notable that estimates of the prevalence of SARS-COV-2-associated PH from the developing world nations (particularly sub-Saharan Africa), are scant and may be difficult to assess because of the high incidence of disorders that may confound the accurate diagnosis of SARS-COV-2-associated PH, such as bronchiectasis, *tuberculosis*, and even schistosomiasis. The true prevalence of PH in SARS-COV-2 patients is likely to vary according to the patterns of regional differences including but not limited to differences in cultural, environmental, and genetic factors, as well as methodological issues regarding the echocardiographic cut-off criteria.

Nevertheless, careful analysis of the published data suggests that the estimated prevalence of SARS-COV-2 associated PH ranges from 12% to 22% globally. Despite this, most studies involve only symptomatic patients, thereby introducing potential selection bias to these estimates and underestimating the magnitude of the disease.

## 7 Clinical diagnosis and management of SARS-COV-2-associated pulmonary hypertension

### 7.1 Diagnostic approach and challenges

Early diagnosis of SARS-COV-2-Associated PH remains a significant clinical challenge as no symptoms have been clearly linked to PH, and they may not differ from other types of PH, such as progressive dyspnea, chest discomfort, dizziness, and syncope. A high index of suspicion and routine screening for SARS-COV-2-Associated PH after SARS-COV-2 infection may thus be essential to facilitate the early detection of patients at risk after SARS-COV-2 infection.

The clinical diagnostic approach to patients with a history of SARS-COV-2 infection and suspected PH should be similar to that used to diagnose PAH in the absence of SARS-COV-2 infection (Galiè et al., 2016). However, in developing countries (particularly sub-Saharan Africa), where cardiac catheterization laboratories may not be unavailable, a possibility of PH when patients with a history of SARS-COV-2 infection present with suggestive symptoms should be considered. Echocardiography remains useful; however, an elevated pulmonary artery systolic pressure should not be considered diagnostic unless there is concomitant evidence of significant right ventricular dysfunction.

### 7.2 Pre-existing co-morbidities

It is well established that certain co-morbidities pose a greater risk for the development of severe COVID-19 and hospital admission. Patients with cardiovascular disease are at greater risk for severe disease, mortality and COVID-19 related complications (Honardoost et al., 2021; Hobohm et al., 2022). It remains a possibility that a number of patients with undiagnosed PH are being admitted with formal evidence of PH found on further testing after the development of COVID-19. This introduces a degree of bias that has not been addressed in the latest studies investigating SARS-COV-2 associated PH. Clinically differentiating pre-existing undiagnosed PH from SARS-COV-2 Associated PH remains a conundrum which requires further robust investigation and research. Although the true prevalence of this condition remains to be accurately elucidated, the mechanisms by which SARS-COV-2 can promote the development of PH still remains a unique, independent and often ill-recognised entity.

### 7.3 Treatment approaches

Treatment of COVID-19 should proceed as per the latest local and international guidelines irrespective of the presence of PAH, however, the addition of medications targeting PH such as endothelin receptor antagonists, phosphodiesterase five inhibitors and prostacyclin may be considered. Although few authors have reported that they treated their patients with SARS-COV-2-associated PH with PAH-specific therapy (Raval et al., 2021), none of these drugs have been studied in sufficiently powered randomized, clinical trials in this patient population. Further studies regarding the effects of these medications for the treatment of SARS-COV-2 Associated PH, especially in the context of varying severities of COVID-19, are needed.

## 8 Classification and PAH-specific therapy in SARS-COV-2-associated pulmonary hypertension

The general goal of clinical classification of PH is to categorise clinical disorders associated with PH based on similar etiologies, pathobiological mechanisms, clinical presentation, haemodynamic characteristics, and therapeutic management (Simonneau et al., 2019). According to the World Health Organization (WHO), PH is classified into five groups based on different causes including Group 1: Pulmonary Arterial Hypertension (PAH); Group 2: PH due to left-sided heart disease; Group 3: PH due to chronic lung diseases, hypoxia or both; PH due to CTEPH and other pulmonary obstructive processes; Group 5: PH due to multifactorial mechanisms or unclear mechanisms.

**FIGURE 5:**
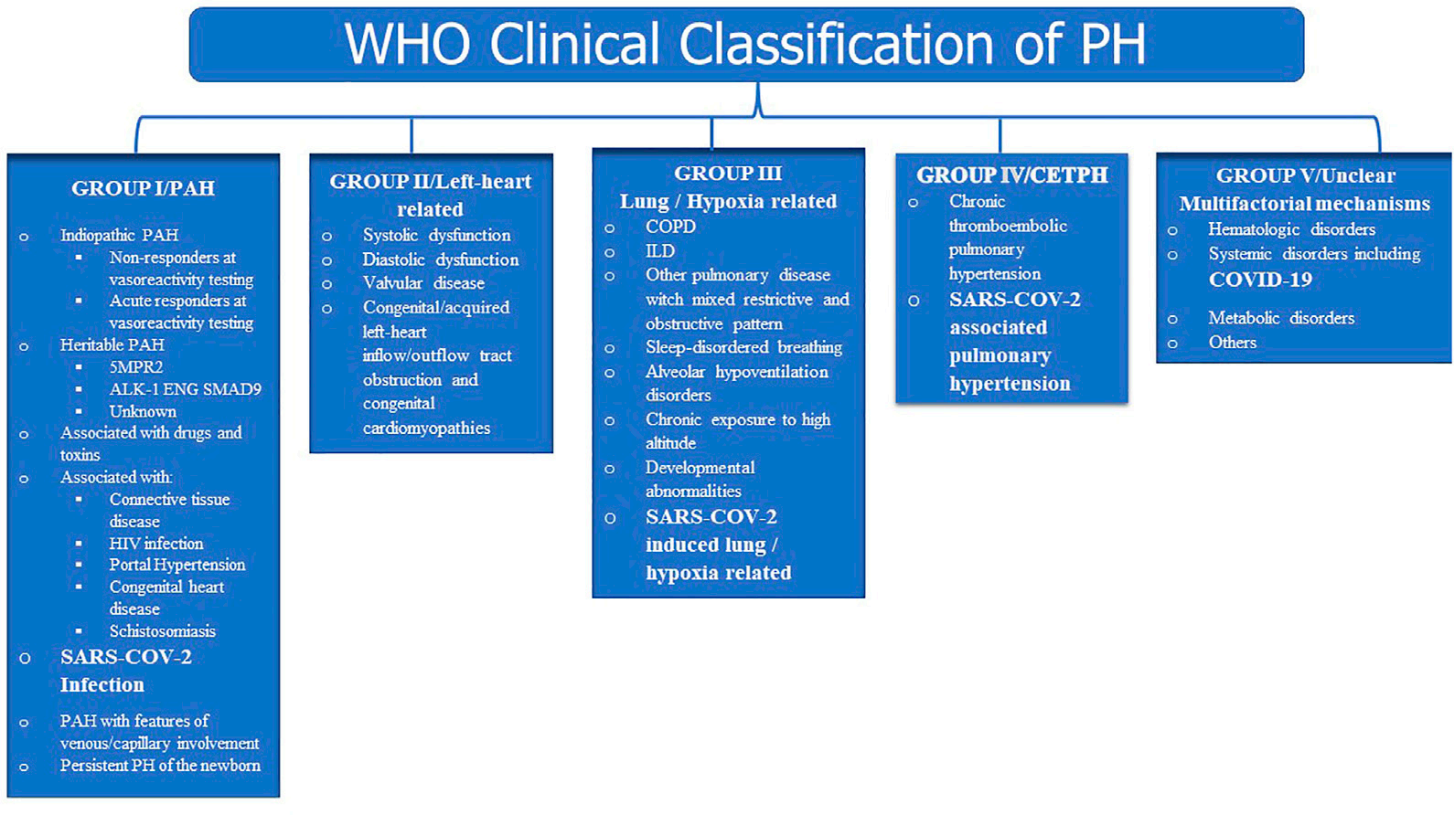
Suggested clinical classification.

The pathobiology of SARS-COV-2-Associated could be multifactorial due to extensive pulmonary parenchymal damage (induced by interstitial and alveolar inflammation) in the form of interstitial lung disease or significant hypoxia (group 3), associated to pulmonary microvascular injury (due to thromboembolic processes, endothelial injury, or, at least, hypoxic vasoconstriction) (group 4), associated to direct damage of the vessel wall mediated by activation of complement pathways (group 1) or due to multiple combined mechanisms including inflammation, oxidative stress, DNA damage, mitochondrial dysfunction and mechanical ventilation (Group 5) (Magro et al., 2020; Potus et al., 2020; van Dongen et al., 2020; Tudoran et al., 2021; Cueto-Robledo et al., 2022). As a result, we propose a revised WHO clinical classification of PH as illustrated in Figure 5.

## 9 Prevention of SARS-COV-2 associated pulmonary hypertension

Although SARS-COV-2 Associated PH remains a relatively new clinical entity and thus the evidence base for optimal prevention strategies are lacking, the most effective strategies by which to prevent SARS-COV-2 Associated PH also is to prevent SARS-COV-2 infection and COVID-19 (such as vaccination, masking, social distancing, hand hygiene). It is likely that any measure that decreases the incidence of acute SARS-COV-2 infection will in turn decrease the incidence and probably, the severity of SARS-COV-2 Associated PH. As has been highlighted above, the development of PH seems to occur regardless of severity of COVID-19 and has been demonstrated in patients that contracted the virus but were wholly asymptomatic.

Although adherence to national vaccination programs and ensuring uptake of booster doses may be considered as the greatest strategy to achieve this, the type of vaccines used may be relevant. As previously mentioned, even transient exposure of picomolar concentrations of S1 protein alone is sufficient to trigger cell growth signalling *via* the MEK/ERK pathway in human pulmonary vascular smooth muscle and endothelial cells and (Suzuki et al., 2021)this can promote hyperplasia and/or hypertrophy of the pulmonary vasculature as MEK phosphorylation has been associated with de-differentiation, nuclear activation and proliferation of pulmonary vascular smooth muscle cells in pulmonary hypertension (Xing et al., 2019; Suzuki et al., 2021). These observations raise the intriguing question of whether the transmembrane-anchored or free circulating spike protein S1 subunit antigen for vaccination may predispose vaccinated subjects to the development of a deadly disease, pulmonary arterial hypertension (PAH) in the future. Whether the risk of PAH is greater following COVID-19 than after mRNA vaccination requires further investigation. Significant further research is thus needed to develop an approach to prevent the development of PH in patients already infected with SARS-COV-2.

## 10 Conclusion

SARS-COV-2 should be considered one of the causative factors of PH as patients with SARS-COV-2 infection are significantly more susceptible to developing PH in comparison with the general population. The pathobiology of SARS-COV-2 -associated PH is still far from being fully understood but is likely multifactorial with SARS-COV-2 viral proteins being candidate contributors to vascular injury and pulmonary arteriole remodelling. Other relevant factors include hypoxia, increased inflammation, and the activation of the complement system.

Increased insight into this clinical condition is of utmost importance and additional data are required to further develop *our understanding* of mechanisms underpinning the pathobiology of SARS-COV-2-associated PH to identify disease-specific therapies for this complex and morbid type of pulmonary vascular disease.

After an acute SARS-COV-2 infection, there are clinical and viral risk factors (including SARS COV-2 Viral proteins, mRNA vaccins, exposure and recurrent exposure to SARS-COV-2 virus, other infections, toxic drugs and genetics), mediators of thrombus remodeling [i.e., IL-8, transforming growth factor (TGF)-β], hypoxia, immunological and inflammatory mediators, and defects in fibrinolysis that combine to determine whether the thrombotic material resolves or becomes a collagen-rich vascular scar. About 12%–22% of patients will have pulmonary hypertension after acute SARS-COV-2 infection, and probably a significant of these patients will have a resolution or persistent elevation of pulmonary artery pressures. IP-10 = IFN-γ–inducible protein-10.

Although the effects of the co-infection with SARS-COV-2 on pulmonary vascular disease development are still unknown; it remains to be determined whether the overlap in cytokines profiling patterns in SARS-COV-2, HIV and Schistosomiasis promotes synergistically PAH development in coinfected patients. Co-exposure of these pathogens may potentiate pulmonary vascular disease resulting in different remodeling phenotypes as the dysregulation of inflammatory responses induced by one may influence the pattern of pulmonary vascular remodeling mediated by the other.

The exact underlying pathobiology by which to account for pulmonary vascular disease in SARS-COV-2 infected individuals remains uncertain. Evidence suggests SARS-COV-2 viral proteins (mainly S1 spike protein) may contribute to endothelial cell injury and initiate pulmonary vascular remodeling *via* a fast activation of MEK/ERK pathway in human pulmonary vascular smooth muscle and endothelial cells. SARS-COV-2 spike protein is reported to increase intracellular reactive oxygen species (ROS) levels, which, in turn, may trigger the autophagic and inflammatory responses as well as programmed cell death or apoptosis in infected pulmonary vascular endothelial cells. SARS-COV-2 membrane glycoprotein M with the assistance of the nucleocapsid protein N may induce caspase-mediated signaling pathways apoptosis via interacting with PDK1 and inhibiting the activation of PDK1-PKB/Akt signaling. In addition, SARS-COV-2 viral proteins may interact with different types of cell surface receptors of pulmonary vasculature to induce upregulation of the p38 MAPK pathway, VCAM-1 expression, nuclear factor–kappa B (NF-κB) translocation *via* the reduction of the dual-specificity phosphatases, and up-regulation of the hypoxia-inducible factor–1α (HIF-1), which, collectively are implicated in the pathobiology of pulmonary vascular remodeling and dysfunction. SARS-COV-2 viral proteins may also interact with different other types of cell surface receptors to increase local concentrations of specific inflammatory cytokines known to mediate pulmonary vascular remodeling such as platelet-derived growth factors (PDGFs), vascular endothelial growth factor (VEGF), Interferon gamma inducible protein 10 kD (IP-10) and fibroblast growth factor-2 (FGF2). Additionally, SARS-COV-2 viral proteins may also induce the sustained elevation of the potent vasoconstrictor endothelin-1 in pulmonary vascular endothelial cells. Apoptotic cell clearance by the professional phagocytes (by phagocytosis), done as part of the long-term maintenance of tissue homeostasis, may in fact trigger the release of further cytokines and growth factors, which, collectively, may promote excessive and complex pulmonary vascular cell proliferation and remodeling, and PH. SARS-COV-2-mediated release of cytokines such as IL-6, or increased levels of the endogenous competitive inhibitor of endothelial nitric oxide synthase, asymmetrical dimethylarginine (ADMA), may also promotes endothelial dysfunction and vascular smooth muscle cell proliferation and, thus, contribute to SARS-COV-2-associated PH. In addition, SARS-COV-2 viral proteins may modulate monocyte chemotactic protein-1 (MCP-1) and the release of IL-2, which may promote pulmonary vascular remodeling. Circulating chemokines may also promote SARS-COV-2-associated PH *via* an interaction with pulmonary vascular endothelial cell receptors.

These hypothetical observations support the concept that SARS-COV-2 - PAH may be a “multiple hit” phenomenon and that recurrent asymptomatic or mild COVID-19 as well as toxic drugs-induced endothelial cell injury may be some of such insults. Identifying these risk factors as well as understanding the multiple hits in the pathogenesis of the SARS-COV-2-PAH will help identify high-risk individuals and optimize medical management for this deadly disease.
